# Cortical atrophy patterns in myelin oligodendrocyte glycoprotein antibody‐associated disease

**DOI:** 10.1002/acn3.52137

**Published:** 2024-07-25

**Authors:** Ruth Schneider, Ann‐Kathrin Kogel, Theodoros Ladopoulos, Nadine Siems, Britta Krieger, Barbara Bellenberg, Ralf Gold, Ilya Ayzenberg, Carsten Lukas

**Affiliations:** ^1^ Department of Neurology, St. Josef Hospital Ruhr University Bochum Bochum Germany; ^2^ Institute of Neuroradiology, St. Josef Hospital Ruhr University Bochum Bochum Germany

## Abstract

**Objectives:**

Global brain volume changes in patients with myelin oligodendrocyte glycoprotein antibody‐associated disease compared with healthy controls (HC) could be revealed by magnetic resonance imaging, but specific atrophy patterns of cortical structures and relation to cognitive impairment are not yet comprehensively known. Thus, we aimed to investigate cortical thickness differences in patients with myelin oligodendrocyte glycoprotein antibody‐associated disease compared with HC.

**Methods:**

3‐Tesla brain magnetic resonance imaging was performed in 23 patients with myelin oligodendrocyte glycoprotein antibody‐associated disease and 49 HC for voxel‐wise group comparisons and neuropsychological testing in patients. Surface‐based morphometry with region of interest‐based surface analysis and region of interest‐based extraction of cortical thickness was performed in patients compared with HC and in patient subgroups with and without cognitive impairment.

**Results:**

Comparing patients with myelin oligodendrocyte glycoprotein antibody‐associated disease with HC, exploratory surface‐based morphometry demonstrated cortical volume reduction in pericalcarine and lingual cortical regions. Region of interest‐based surface analysis specified reduced cortical thickness in the adjacent pericalcarine and orbitofrontal regions in myelin oligodendrocyte glycoprotein antibody‐associated disease, as well as reduced temporal cortical thickness in patients with cognitive impairment (*n* = 10). Patients without cognitive impairment (*n* = 13) showed only circumscribed cortical brain volume loss compared with HC in the pericalcarine region.

**Interpretation:**

In conclusion, cortical atrophy in myelin oligodendrocyte glycoprotein antibody‐associated disease was characterized by cortical thickness reduction in the adjacent pericalcarine and orbitofrontal regions, with a tendency of temporal thickness reduction in cognitively impaired patients.

## Introduction

Myelin oligodendrocyte glycoprotein (MOG) antibody‐associated disease (MOGAD) is an inflammatory demyelinating disease of the central nervous system (CNS), in which immunoglobulin G (IgG) antibodies against MOG, which is expressed on myelin sheaths and oligodendrocytes, can be detected.^1^ MOGAD can show very different manifestations in magnetic resonance imaging (MRI), depending on its characterization during an acute relapse or in the postrelapse state.^2^ Beyond obvious MRI changes during relapse, with extensive T2 lesions in brain, spine, and/or orbits,^3^, ^4^ MRI during nonacute disease stages are relevant in terms of differentiating MOGAD from other demyelinating CNS diseases, and for characterizing on‐going pathophysiological processes.^5^

Cortical pathology is particularly relevant in MOGAD. Cortical demyelination in this disease is frequently found on histopathological investigations^6^; in contrast to multiple sclerosis (MS), intracortical, rather than leukocortical, demyelinated lesions dominate. Relevant effects on cortical gray matter were also suggested by diffuse cortical MRI signal changes, which can be observed during the clinical course of MOGAD‐induced encephalitis with epileptic seizures.^7^

The detection of cortical lesions in MOGAD also illustrates their relevance with regard to the differentiation from neuromyelitis optica spectrum disorders in which cortical lesions do not occure.^8^ Duan et al. recently demonstrated the presence of cortical and juxtacortical lesions in 68% of patients with MOGAD and verified both cortical and subcortical gray matter atrophy in MOGAD, similar to a pattern seen in MS.^9^ A higher number of relapses early after MOGAD diagnosis has been associated with accelerated total brain volume loss; thus, early gray matter affection resulting in regional atrophy appears relevant to the disease course.^10^ The relevance of deep gray matter affection in MOGAD based on volumetric MRI investigation is reported in several studies.^11^, ^12^ Regarding regional patterns of cortical gray matter atrophy pattern, Zhuo et al. showed gray matter atrophy in the frontal and temporal lobes in MOGAD compared with controls. In that study, gray matter atrophy was also associated with clinical disability and cognitive impairment.^13^ The relevance of cognitive impairment has been investigated in both pediatric‐onset relapsing MOGAD and, recently, adults, revealing an association between cognitive impairment and gray matter volume loss.^14^, ^15^ In view of the fact that a clear characterization of cognitive impairment in MOGAD has not been established,^16^ and a multifactorial genesis must be taken into account, cortical gray matter pathology is probably only one component that explains the clinically relevant cognitive deficits in MOGAD.

Thus, the aim herein was to exploratively identify cortical atrophy patterns in patients with MOGAD compared to healthy control (HC) and to verify regional cortical thickness reduction in patient subgroups with cognitive impairment (MOG_Ci), and those with preserved cognition (MOG_Cp), compared with HCs, using surface‐based morphometry (SBM) MRI analysis.

## Materials and Methods

### Participants and study design

Using international diagnostic recommendations, 23 adult patients with suggestive clinical phenotype and positive MOG‐IgG titer, as a part of a prospective MOGAD cohort, were included in this MRI study.^17^ Inclusion criteria were independent of clinical phenotype and were based on patient availability and consent to participate in a research MRI examination at the time of recruitment. Patients received neuropsychological testing and MRI investigation at least 3 months after a previous relapse event. Additionally, 49 age‐matched HC underwent MRI. All subjects gave their written informed consent for inclusion before they participated in the study. The study was conducted in accordance with the Declaration of Helsinki, and the protocol was approved by Institutional Review Board of the Ruhr‐University Bochum (#15–5534). Anonymized data used for this study are available from the corresponding author upon reasonable request.

### Neuropsychological examination

Patients with MOGAD were examined by two certified neuropsychologists with a standardized test battery in German language commonly used in our center. This test battery includes examinations of attention (subtest “Alertness” of the Test of Attentional Performance [TAP] measuring “tonic” and “phasic” alertness),^18^ mental flexibility as a part of executive functions (subtest “phonematic category change” of the Regensburger Word fluency test [RWT]),^19^ verbal fluency (subtest “phonematic fluency” of the RWT), visual memory (subtest “visual reproduction” of the revised Wechsler Memory Scale [WMS‐R]),^20^ mental rotation as an visuospatial function (subtest 7 of the Performance test system[LPS 7]),^21^ verbal Working and short‐term Memory (subtest “Digit Span forwards and backwards” of the WMS‐R), and Verbal Memory (Verbal learning and memory test, [VLMT]).^22^ Cognitive impairment was defined as performance <16th percentile, using demographically adjusted scores, in ≥2 cognitive tests representing different cognitive domains. Participants also completed the self‐reported Fatigue Scale for Motor and Cognitive Functions (FSMC).^23^

### Magnetic resonance imaging

Imaging was performed with a single 3Tesla (T) scanner (Achieva Philips, Best, The Netherlands) using a standardized imaging protocol, with isotropic 3D fluid attenuated inversion recovery (FLAIR) sequence for lesion quantification (170 sagittal slices; field of view [FOV] 240 mm; resolution 1 × 1 × 1 mm3; TR/TE/inversion time 4800/286/1650 ms; turbo factor 182; acquisition time 6 min 30 s) and structural isotropic T1‐weighted 3D sequence (T1 fast field echo; 180 sagittal slices; FOV 240 mm × 240 mm; voxel size: 1 × 1 × 1 mm; TR, TE, TI/ms: 10/4.6/1000; flip angle 8°; turbo factor 164; acquisition time 6 min) for volumetric analysis.

### Volumetric and surface‐based analysis

Volumetric and surface‐based processing was performed using Statistical Parametric Mapping software (SPM 12, revision 7771) and Computational Anatomy Toolbox 12 (CAT12, version 12.8 (r1932), Structural Brain Imaging Group, University of Jena)^24^ run with MATLAB version R2018b (Mathworks, Inc., Massachusetts, USA). Prior to volumetric analysis, lesion segmentation toolbox (LST toolbox, version 3.0.0) for SPM12 was used for lesion segmentation and filling in order to correct 3D‐T1 weighted images for white matter lesions.^25^ Data preprocessing was performed using the default preprocessing steps of basic voxel‐based morphometry analyses with CAT12 for normalizing, segmenting, and smoothing the data.^24^ Preprocessing provided estimations of gray matter and white matter volumes, total intracranial volume (TIV, in mL), and average global brain cortical thickness in mm. Gray matter and white matter were calculated as percentages of TIV.

### SBM group comparison

Being aware of cortical thickness estimation, giving an indication of a relevant difference in cortical thickness in between HC and patients as well as in between patients with and without cognitive impairment, exploratory SBM was performed as an additional step in CAT 12 after cortical thickness estimation, in the context of data segmentation during the preprocessing steps, to verify the signal of disease‐related cortical thickness differences. We used 15 mm kernels as the smoothing filter size, in full width at half maximum for thickness data. For statistical analyses of surface measures, we used a second‐level model. Imaging data were statistically analyzed by ANOVA, for SBM‐estimated cortical thickness. Against the background of the age‐matched cohorts and thus an equally advanced atrophy development to be expected with regard to aging,^16^ disease duration within the patient cohorts and sex for both groups were included as covariates in the statistical models. Because of the small overall number of patients with MOGAD, and especially the subgroups with cognitive impairment (MOG_Ci *n* = 10) and preserved cognition (MOG_Cp *n* = 13), we performed global SBM analyses without family wise error (FWE) corrections, to detect a trend in local cortical thickness reduction patterns. Statistically significant clusters of cortical thickness differences based on group comparisons were localized using Desikan–Killiany 40 (DK40) atlas as a template to extract cluster sizes and proportional overlaps with atlas regions. SBM group comparisons were performed separately for all patients with MOGAD compared with HC, for MOG_Ci compared with HC, for MOG_Ci compared with MOG_Cp, and for MOG_Cp compared with HC.

### ROI‐based between‐group surface analysis

To verify trends in between‐group differences in the explorative uncorrected SBM analysis, we performed a region of interest (ROI)‐based analysis of cortical thickness with surface‐based atlas maps.^24^ We used the DK40 atlas to define anatomical ROI, which provides 34 cortical regions in the left and right hemispheres. Results of surface‐based ROI analyses were further analyzed with a second‐level ANOVA model with a *p*‐value of 0.05, corrected for multiple comparisons with false discovery rate (FDR) and Holm–Bonferroni, for left and right hemispheres. Group comparison designs and covariates were the same as the SBM group comparison, including sex and disease duration (for patients) covariates. We thus performed the ROI‐based group comparisons separately for all patients with MOGAD compared with HC, MOG_Ci compared with HC, MOG_Cp compared with HC, and MOG_Ci compared with MOG_Cp.

### Association of Global and Regional Cortical Thickness with cognitive tasks

For regions with significant cortical volume reduction on ROI‐based group comparisons, we used the ROI‐based surface value extraction function of CAT 12 to estimate the average cortical thickness in each ROI for all patients with MOGAD and for HC. For those ROI in which significantly reduced cortical thickness was detected in the comparison between MOG_Ci and HC, or between MOG_Ci and MOG_Cp, we further investigated the associations of average cortical thickness values with selected cognitive tests. We chose tasks for which a correlation between the assessed neurocognitive function and corresponding brain region might be assumed based on known neuroanatomic‐functional relations. Thus, we investigated the association of FSMC (total, motor, and cognitive) with global mean cortical thickness. We also analyzed the association of visual memory performance (WMS‐R visual reproduction) and VLMT with cortical thickness of the left inferior temporal and middle temporal regions, and the left temporal pole.

### Statistics

SPSS (IBM) software (version 26) was used for further statistical analyses. Group comparisons of global brain volumes were performed using univariate ANOVA, with age and sex as confounders, and tests for significant group comparisons were made with post hoc pairwise tests adjusted for multiple comparisons using Sidak correction. We used Spearman correlations to analyze the associations between regional cortical thickness and cognitive tasks.

## Results

### Demographic, clinical, and global MRI data

Demographic and global brain MRI data for all patients with MOGAD, subgroups, and HC are presented in Table 1. Because of the significant sex difference between MOGAD and HC, sex was used as a covariate in all volumetric analyses. Disease duration was also used as a covariate, because of the significantly longer disease duration in MOG_Cp compared with MOG_Ci. No significant global gray matter volume differences were found between patients with MOGAD and HC, while average cortical thickness was lower in MOGAD compared with HC and in MOG_Ci compared with HC. Global white matter volume was reduced in MOGAD compared with HC. In our cohort, 10 patients (43%) demonstrated cognitive impairment. Sixteen patients (70%) had lower scores (<16th percentile) in one domain only. Most prevalent were deficits in mental flexibility (33%), verbal working memory (30%), and attention (24%). With regard to clinical presentation, an acute demyelinating encephalomyelitis (ADEM)/ADEM‐like episode (4 out of 5 patients), a non‐ADEM brain relapse (3 out of 3 patients) and cortical encephalitis were identified proportionally more frequently in cognitively impaired patients with MOGAD. Additional neuropsychological and clinical data are provided in the supplement (Tables S1 and S2).

**Table 1:** Demographics and global brain and lesion volumes for all patients with MOGAD, subgroups, and HC.

Mean ± SD	All patients with MOGAD	MOG_Ci	MOG_Cp	HC
Number	*n* = 23	*n* = 10	*n* = 13	*n* = 49
(Female %)	(73.9%)	(70%)	(76.9%)	(53%)^#^
Age [years]	33.3 ± 12.8	28.3 ± 11.6	37.2 ± 12.7 n.s.	31.4 ± 7.3
Disease duration^@^ [months]	56.3 ± 84.1	24.7 ± 49.5	80.5 ± 98.3 *p* < 0.001^c^	–
Lesion volume [mL]	2.67 ± 6.1	2.74 ± 4.3 n.s.^§^	2.65 ± 7.5	–
Gray matter [%]/TIV	45.39 ± 3.0	45.6 ± 3.51	45.22 ± 2.67	45.52 ± 2.36
White matter [%]/TIV	34.11 ± 2.53* *p* = 0.028^a^	34.54 ± 2.85	33.78 ± 2.32	35.3 ± 1.8*
Cortical thickness [mm]	2.41 ± 0.1* *p* = 0.03^a^	2.39 ± 0.1* *p* = 0.020^c^	2.43 ± 0.1*	2.47 ± 0.1 *p* = 0.024^b^

HC, healthy controls; MOG_Ci, cognitive impaired patients with MOGAD; MOG_Cp, cognitive preserved patients with MOGAD; MOGAD, myelin oligodendrocyte glycoprotein antibody‐associated disease; SD, standard deviation; TIV, total intracranial volume.

^a^ Significant MOGAD and HC difference.

^b^ Significant MOG_Ci and MOG_Cp and HC difference.

^c^ Significant MOGAD subgroup difference.

* *p* < 0.05 for between‐group differences using univariate ANOVA, with age and sex as confounders; post hoc pairwise tests adjusted for multiple comparisons using Sidak correction.

^@^ Disease duration refers to the time of the MRI.

^#^ Significant Pearson's chi‐square test (*χ* ^2^) test for MOGAD/MOGAD subgroups/HC sex difference.

^§^ Group comparison between MOG_Ci and MOG_Cp using Mann–Whitney *U* test (*U* = 44.5, *Z* = −1.272, *p* = 0.213).

### SBM analysis of patients with MOGAD and HC

Uncorrected surface‐based group comparisons for regions with significantly reduced cortical thickness values, with an affected cluster size >30 voxels, are summarized in Table 2. The attributed atlas regions are shown for atlas regions and clusters having an overlap >20%.

**Table 2:** Reduced cortical gray matter regions for group comparisons between MOGAD, MOG_Ci, MOG_Cp, and HC, identified by surface‐based morphometry analyses.

Cortical atlas regions	MOGAD versus HC			MOG_Ci versus HC			MOG_Ci versus MOG_Cp		
	Cluster size	Overlap (%)	*p*‐value	Cluster size	Overlap (%)	*p*‐value	Cluster size	Overlap (%)	*p*‐value
Left hemisphere									
Lingual	255	54	0.00002	215	59	0.00002			
Pericalcarine		46			40				
Superior frontal	59	59	0.00041						
Posterior cingulate		22							
Paracentral		19							
Lingual	58	55	0.00036						
Parahippocampal		43							
Temporal pole							31	100	0.00024
Right hemisphere									
Pericalcarine	128	66	0.00001	100	76	0.00005			
Lingual		15			24				
Middle temporal	69	100	0.00007						
Medial orbitofrontal	58	100	0.00003	66	97	0.00001			
Postcentral	46	76	0.00047						
Supramarginal		24							
Inferior temporal	46	100	0.00049						
Superior temporal							44	100	0.00008

Uncorrected results with a cluster size >30 voxels and an overlap with DK40 atlas regions of more than 20% are reported.

HC, healthy controls; MOG_Ci, cognitive impaired patients with MOGAD; MOG_Cp, cognitive preserved patients with MOGAD; MOGAD, myelin oligodendrocyte glycoprotein antibody‐associated disease.

Patients with MOGAD compared with HC demonstrated the greatest cortical thickness reductions in the lingual and pericalcarine regions of the left and right hemispheres. A concordant effect was also found in MOG_Ci compared with HC in the lingual and pericalcarine regions of the left and right hemispheres. Comparing MOG_Cp with HC (not shown) showed no cortical thickness reduction affecting a relevant cluster size of >30 voxels.

Comparing MOG_Ci with MOG_Cp, a further notable cortical thickness reduction was shown in temporal cortical regions, particularly in the temporal pole of the left hemisphere and in the superior temporal area of the right hemisphere.

### Between‐group ROI‐based surface analysis

**Figure 1:**
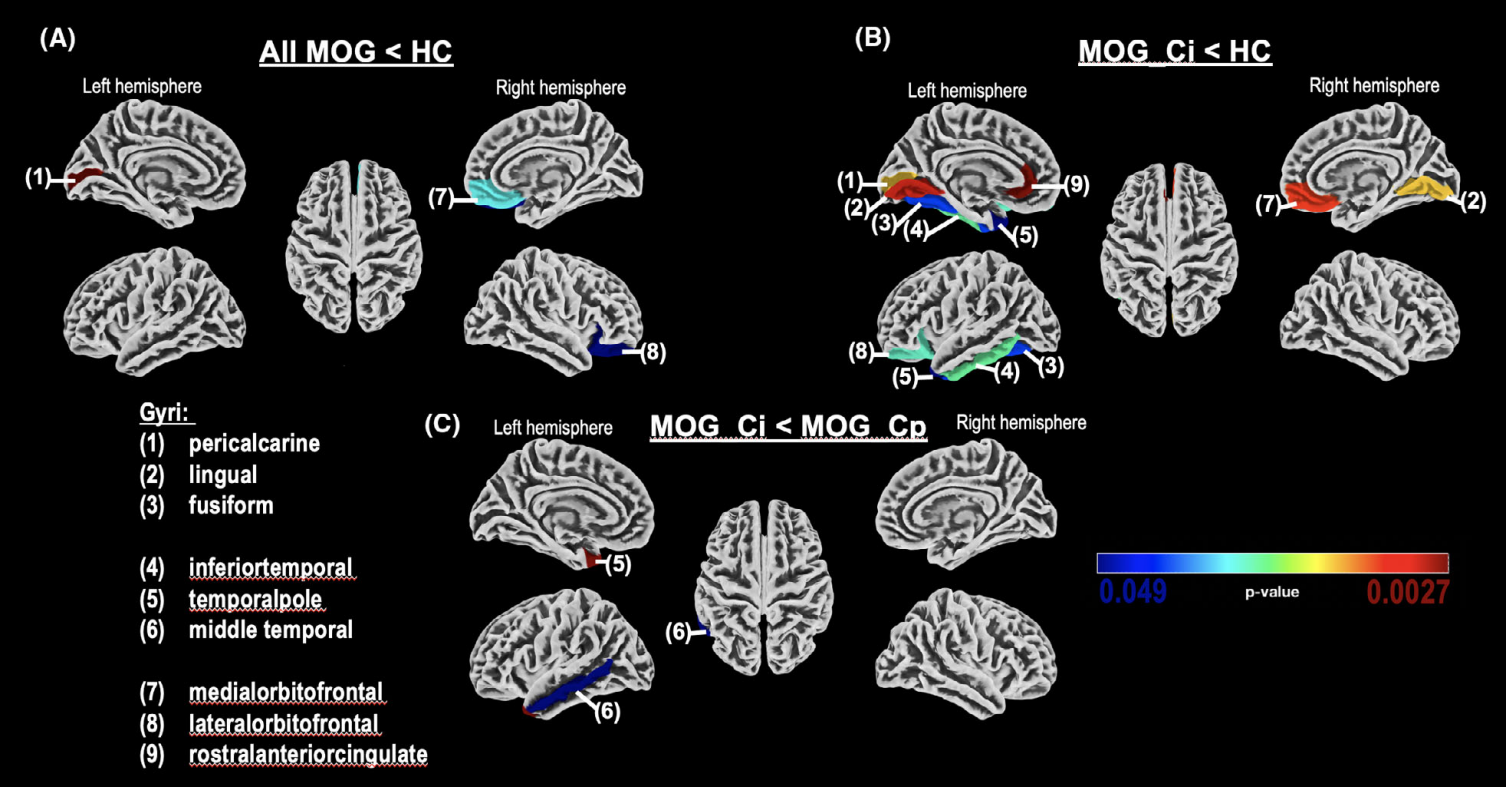
ROI‐based surface analysis between groups with color‐coded significantly reduced cortical regions: (A) Significant reduced CT in MOGAD group compared with HC; (B) Significant reduced CT in MOG_Ci group compared with HC; (C) Significant reduced cortical thickness in MOG_Ci compared with MOG_Cp. Color‐coded *p*‐values in the rank between 0.049 (blue) and 0.0027 (red).

The ROI‐based surface analysis identified significantly reduced cortical thickness between groups within DK40‐defined anatomical regions (Fig. 1). Regions of significant group differences identified with the Holm–Bonferroni and FDR corrections were consistent (Table S3); however, FDR correction identified a few additional significant regions. Ultimately, identified regions were congruent, so the results are presented in Table 3 for the more stringent Holm–Bonferroni. To demonstrate the congruent results, both uncorrected and with both FDR and Holm Bonferroni corrected, results for the between‐group comparisons of patients with MOGAD and HC are in the Supplementary Material.

**Table 3:** Regions with significantly reduced cortical thickness detected by ROI‐based surface analysis using Bonferroni correction for group comparisons between patients with MOGAD and HC using Holm–Bonferroni correction for multiple comparisons.

All_MOGAD versus HC		MOG_Ci versus HC		MOG_Ci versus MOG_Cp		MOG_Cp versus HC	
Atlas region	*p*‐value	Atlas region	*p*‐value	Atlas region	*p*‐value	Atlas region	*p*‐value
Left hemisphere (*p* < 0.05, Holm–Bonferroni corrected)							
Pericalcarine	0.010456	Pericalcarine	0.010499			Pericalcarine	0.044272
		Lingual	0.006033				
		Fusiform	0.029589				
		Rostral anterior cingulate	0.005156				
		Inferior temporal	0.016451				
		Temporal pole	0.045035	Temporal pole	0.002665		
		Lateral orbitofrontal	0.018049	Middle temporal	0.048611		
Right hemisphere (*p* < 0.05, Holm–Bonferroni corrected)							
Medial orbitofrontal	0.031230	Medial orbitofrontal	0.007730				
Lateral orbitofrontal	0.046875	Lingual	0.010456				

HC, healthy controls; MOG_Ci, cognitive impaired patients with MOGAD; MOG_Cp, cognitive preserved patients with MOGAD; MOGAD, myelin oligodendrocyte glycoprotein antibody‐associated disease; ROI, region of interest.

Cortical thickness in patients with MOGAD compared with HC was significantly reduced in the pericalcarine region, especially in the left hemisphere. With FDR correction (see Supplement), there was also a significantly reduced thickness in the pericalcarine region of the right hemisphere, the adjacent left lingual, and the right and left fusiform regions. There was also a thickness reduction in the bilateral orbitofrontal regions in MOGAD, which was verified with Holm–Bonferroni correction in the medial and lateral orbitofrontal cortices of the right hemisphere.

Comparing MOG_Ci with HC, the contiguous regions of the lingual, pericalcarine, and fusiform cortices of the left hemisphere demonstrated significantly reduced cortical thickness in MOG_Ci. Lingual cortical thickness was also significantly reduced in MOG_Ci in the right hemisphere. The rostral anterior cingulate cortex was significantly reduced exclusively in the left hemisphere. Cortical thickness of the temporal regions (including temporal pole and inferior temporal cortex) of the left hemisphere and the orbitofrontal regions were also significantly reduced. The middle temporal cortex was significantly reduced comparing MOG_Ci with HC with FDR correction but fell below the significant threshold with Holm–Bonferroni correction.

Direct comparison between MOG_Ci and MOG_Cp revealed reduced cortical thickness in the left temporal pole and the middle temporal cortex in MOG_Ci. Comparing MOG_Cp with HC, only the pericalcarine region was reduced in MOG_Cp, with relatively weak significance.

### Associations between regional cortical thickness volumes and neuropsychological outcomes

**Figure 2:**
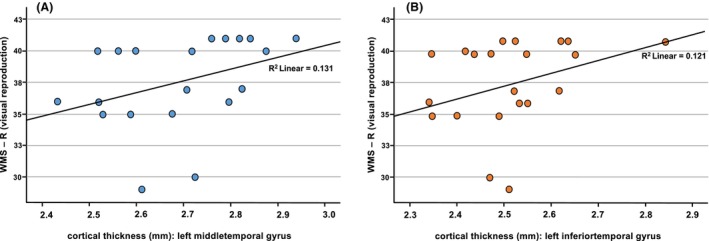
Correlations between cortical thickness of left inferior temporal and middle temporal regions with visual reproduction (WMS‐R).

Association analyses among patients with MOGAD revealed significant correlations between visual memory performance (WMS‐R) and cortical thickness of the left inferior temporal (*p* = 0.031) and middle temporal (*p* = 0.027) regions (Fig. 2). There were no significant correlations between cortical thickness and verbal memory, except between the left temporal pole (*p* = 0.028) and one subtest (VLMT D5). Nor were there any significant associations between mean cortical thickness and fatigue scores.

## Discussion

Herein, in patients with MOGAD, global cortical atrophy was revealed as significant average cortical thickness reduction compared with HC, driven by the accelerated cortical thickness reduction in patients with cognitive impairment compared with HC. This finding is in line with cortical gray matter reduction in patients with MOGAD compared with HC reported by Zhuo et al.^13^ Cortical volume loss in MOGAD was also reported by Duan et al., who reported lower subcortical gray matter volumes which, for methodological reasons, was not investigated herein.^9^ While global gray matter volume was not significantly reduced compared with HC, presumably due to the small sample size, significant global white matter volume reduction was detected, consistent with the volumetric findings of Rechtman et al.^10^

Based on our results, cognitive impairment was found in 43.5% (10/23) of patients with MOGAD. In inflammatory demyelinating conditions of the central nervous system like in MOGAD and neuromyelitis optica spectrum disorder, patients can experience clinically relevant cognitive impairment, although a characterization of cognitive impairment in MOGAD and its underlying pathology is still pending.^26^, ^27^ It can be assumed that in MOGAD, beside other cognitive‐relevant brain impacts, cortical volume loss is a relevant determinant, considering the high rate of cortical demyelination and lesions in that disease, which are absent in Aquaporin‐4‐positive NMOSD.^6^, ^8^

Global gray matter volume (Gray matter [%]/Total intracranial volume; Table 1) showed no significant group differences comparing MOGAD subgroups or patients with control subjects, while global cortical gray matter thickness was significantly reduced comparing patients with MOGAD and control subjects as well as comparing cognitive impaired and preserved patients. Thus, the most pronounced reduction of cortical thickness in cognitively impaired patients with MOGAD seems to maintain the significant difference of cortical thickness in the total MOGAD group compared to control subjects, which emphasizes the particular relevance of cortical pathology in cognitively impaired patients with MOGAD. That white matter volume was significantly reduced in the entire MOGAD group compared with HC seems to primarily driven by the MOG_Cp group, which had the lowest percentage of white matter from the TIV, suggesting that white matter volumetric changes may be less relevant to cognition in MOGAD. Based on these global volume analyses, we were able to demonstrate that in patients with MOGAD, relevant atrophy of cortical gray matter and white matter compared with HC can be detected, indicating that cortical thickness reduction may be a relevant determinant for cognitively impaired patients with MOGAD. Therefore, an explorative analysis of cortical thickness to identify an indication of underlying atrophy patterns using surface‐based morphometry seems warranted.

Explorative uncorrected surface‐based group comparisons identified cortical volume reductions, especially in adjacent pericalcarine and lingual regions, between MOGAD and HC, and additionally in orbitofrontal and temporal regions when comparing MOG_Ci to both HC and MOG_Cp. To verify this trend, a ROI‐based surface analysis with Holm–Bonferroni correction was used to confirming the results, particularly in the MOG_Ci group. Those patients showed reduced cortical thickness in the adjacent left pericalcarine, lingual, and fusiform regions, and in the left inferior temporal region and temporal pole. Right hemispheric cortical thickness in the medial orbitofrontal and lingual regions were reduced in patients with MOG and CI compared with HC. However, the temporal region seems to indicate the strongest relevance between reduced cortical thickness and cognition and was also significantly reduced when comparing the MOG_Ci and MOG_Cp groups. Considering that cortical pathology is only one component that may be related to cognition, we would like to briefly discuss relevant cortical regions identified by our regional surface‐based morphometry analysis in terms of their association with cognitive functions described in the literature. Based on functional neuroimaging studies, the middle temporal gyrus and inferior temporal gyrus may be involved in several cognitive processes. Specifically, the middle temporal cortex facilitates language and semantic memory processing, and the inferior temporal gyrus is involved in visual perception.^27^, ^28^ The relevance of left‐hemispheric middle temporal gyrus and the inferior temporal gyrus in chronic inflammatory demyelinating disorders, including MS and, to a lesser extent NMOSD, is underlined by structural network analyses.^29^ Therefore, volume reduction of the temporal cortical source regions may be assumed in a disease like MOGAD, which has a degree of cortical pathology similar to MS. Furthermore, cortical thinning in MS, especially of the temporal regions, may be related to cognitive impairment.^30^ If so, this would also be relevant to MOGAD. Our evidence of a relation between cortical thickness of the left inferior temporal and middle temporal region with visual memory performance (WMS‐R visual reproduction) may supports this hypothesis.

The temporal pole, in which cortical thickness reduction was demonstrated by comparing MOG_Ci to both HC and MOG_Cp, has been associated with several high‐level cognitive processes, including visual processing of complex objects, facial recognition, and semantic processing in all modalities.^31^ In MS, temporal pole cortical thickness reduction occurs in more progressive disease types with increased lesion load in the temporal pole.^32^ Therefore, it might be useful to consider including lesions in future analyses, especially the occurrence of intracortical lesions which are more frequent in MOGAD.^6^ Even impacts on frontal cortical regions may be indicated, given the reduced cortical thickness in medial and lateral orbitofrontal regions between HC and both MOGAD and MOG_Ci. These results are in line with those of Zhuo et al., who showed gray matter atrophy in the frontal and temporal lobes in MOGAD compared with HC, and an association with cognitive impairment.^13^ The orbitofrontal cortex, containing olfactory cortical areas as well as secondary taste cortex, also receives information about viewed objects from the temporal lobe cortical visual areas. The integration of this cumulative information and stimuli results in its function in the context of associative learning.^33^ Therefore, joint cortical thickness reduction of the temporal and orbitofrontal regions might have a relevant impact on cognitive impairment in MOGAD. This cumulative evidence indicates that the cortical, especially temporal and possibly even orbitofrontal regions, are particularly affected by cortical atrophy in patients with MOGAD, especially those with cognitive impairment.

Regarding the cortical thickness reduction in the left pericalcarine region, MOG_Ci and MOG_Cp were similarly different from HC, indicating a disease‐related change in cortical thickness based on a mechanism shared by these subgroups. The pericalcarine cortical region, the major primary visual cortical area, is influenced by visual input is known to be altered in MOGAD.^34^, ^35^ The adjacent lingual cortex, the location of the secondary visual cortex, was exclusively reduced in both hemispheres when MOG_Ci were compared with HC. This cortical region's function is strongly related to cognitive performance, as it is a component of the visual attention network and spatial memory.^36^, ^37^ The same applies to the fusiform cortex, which is a part of the human ventral temporal cortex, a key structure for functionally specialized computations of high‐level vision such as face perception, object recognition, and reading.^38^ Thus, it may be concluded that joint reduction of cortical thickness in these three adjacent regions, which are in turn related to visual cognitive functions, might impact cognition in patients with MOGAD.

As described above, cortical thickness was significantly reduced in patients with MOGAD compared with HC, especially in those with cognitive impairment. This suggests that cortical pathology is relevant in MOGAD, consistent with the clinical manifestations of ADEM as the primary encephalitic presentation, and other meningocortical manifestations of MOGAD.^35^ Supported by the proportionally more frequently identified patients with ADEM/ ADEM‐like episode as well as a non‐ADEM brain relapse in our cohort, ADEM itself may be a risk factor for cortical atrophy and associated cognitive impairment in MOGAD.^15^

The main study limitation was the inclusion of a relatively limited sample of patients with MOGAD, especially within the neuropsychological assessment‐based subgroups. Because of this, we were only able to use global, uncorrected surface‐based morphometry analysis to detect a trend in global cortical thickness reduction. To perform a more targeted analysis, we performed between‐group ROI‐based surface analysis, applying both FDR and Holm–Bonferroni correction for multiple comparisons. Nevertheless, the exploratory nature of the study should be emphasized, especially with regard to the association between neuropsychological subtests and the thickness of presumed associated cortical regions. Because surface‐based morphometry analyses focused on neocortical gray matter, no deductions can be made about pathology of the cerebellum or its cortical structure. Cerebellar functions are known to influence cognition in chronic inflammatory CNS disease; thus, the analyses herein may underestimate these influences. Furthermore, surface‐based morphometry analysis does not consider deep gray matter structures, which are also relevant to cognition. Finally, it is important to note that inclusion of patients with MOGAD was performed before establishment of the international criteria for the diagnosis of MOGAD.^39^

In conclusion, this explorative study demonstrates a relevant cortical thickness reduction in patients with MOGAD compared with HC affecting orbitofrontal and pericalcarine regions. Regional cortical analysis confirms cortical thickness reductions in adjacent left pericalcarine region and right orbitofrontal regions. Additionally, cortical thickness reduction tended to be more pronounced in left temporal regions in cognitively impaired patients with MOGAD.

## Author Contributions

RS: Design and conceptualization of the study, analysis of data, drafting of the manuscript; AKK, TL, NS, BK: conceptualization of the study, Acquisition and analysis of data, editing; BB: Design and conceptualization of the study; editing; RG, IA, CL: Design of the study and editing.

## Funding Information

No funding information provided.

## Conflicts of Interest

The authors declare no conflict of interest with the present work. Irrespective of the present work the authors report following conflict s of interest: R.S. has received speaker's honoraria from Bayer HealthCare, Alexion Pharma, Novartis Pharma, and Roche Pharma AG, congress travel support from Merck, Biogen Idec GmbH and has received research scientific grant support from Novartis Pharma. T.L.: has received research scientific grant support from Novartis Pharma. A.K., N.S., B.K., B.B.: nothing to disclose. R.G.: has received compensation for serving as a consultant or speaker from Bayer HealthCare, Biogen Idec, Merck Serono, Novartis, and Teva Neuroscience; he, or the institution he works for, has received research support from Bayer HealthCare, Biogen Idec, Merck Serono, Novartis, and Teva Neuroscience; he has also received honoraria as a Journal Editor from SAGE and Thieme Verlag. I.A.: has received travel grants from Alexion, BMS, Horizon, Roche, Biogen Idec, and Guthy‐Jackson Charitable Foundation, served on scientific advisory boards for Merck, Roche, Alexion, Horizon, and Sanofi and received research support from Diamed and Roche. C.L.: received a research grant by the German Federal Ministry for Education and Research, BMBF, German Competence Network Multiple Sclerosis (KKNMS), grant no.01GI1601I, has received consulting and 415 speaker's honoraria from Biogen Idec, Bayer Schering, Daiichi Sanykyo, Merck Serono, Novartis, 416 Sanofi, Genzyme, and TEVA.

## Supporting information


Table S1.

## Data Availability

The data that support the findings of this study are available from the corresponding author upon reasonable request.
