# Supplementary material for: Cortical atrophy patterns in myelin oligodendrocyte glycoprotein antibody‐associated disease
Source: Ann Clin Transl Neurol. 2024 Jul 25;11(8):2166–75. doi: 10.1002/acn3.52137 (PMC11330211; doi:10.1002/acn3.52137)
Supplement: Supplementary file 1 — Table S1. [file ACN3-11-2166-s001.pdf]

Supplement

Cortical Atrophy Patterns in Patients with MOG Antibody-associated Disease, with and without Cognitive Impairment

Ruth Schneider*[1;2], Ann-Kathrin Kogel[1], Theodoros Ladpoulos[1;2], Nadine Siems[1], Britta Krieger[2], Barbara Bellenberg[2], Ralf Gold[1], Ilya Ayzenberg[1], Carsten Lukas[2],

Results

Additional neuropsychological and clinical data:

The clinical data was collected as part of our local MOGAD cohort. Neuropsychological Examination including subtests representing different cognitive domains are presented as well as clinical data in table S1.

Table S1 Neuropsychological and Clinical Data: Neuropsychological Examination (n= number of available tests), Clinical presentation, Clinical data: MOG-Antibody titer, Interval between clinical attack and MRI/Neuropsychological examination and Interval between corticosteroids and MRI in the entire MOGAD group (n=23) and in MOGAD subgroups (MOGAD_Ci n=10; MOG_CI and MOG_Cp n=13).

Neuropsychological examination

Patients/ Subgroups	TAP tonic alertness n=17	TAP phasic alertness n=17	RWT fluency n=21	RWT mental flexibility/exe cutive function n=21	WMS-R verbal short term memory n=23	WMS-R verbal working memory n=23	VLMT (Dg5) verbal memory n=22	VLMT (Dg7)verbal long term memory n=21	LPS-7mental rotation n=18	WMS-R visual (short term) memory n=19
**ALL MOG**	1 (6%)	4 (24%)	3 (14%)	7 (33%)	5 (22%)	7 (30%)	3 (14%)	2 (10%)	3 (17%)	2 (11%)
**MOG_Ci**	-	1 (4%)	2 (10%)	6 (29%)	5 (22%)	7 (30%)	3 (14%)	2 (10%)	3 (17%)	2 (11%)
**MOG_Cp**	1 (6%)	3 (18%)	1 (4%)	1 (4%)						

Clinical presentation

Patients/ Subgroups	ADEM/ ADEM-like n=23	ON n=23	Myelitis n=23	Cortical Encephalitis n=23	Brainstem/ Cerebellum n=23	"non ADEM brain relapse" n=23
**ALL MOG**	5	13	12	1	3	3
**MOG_Ci**	4	6	5	1	2	3
**MOG_Cp**	1	7	7	-	1	-

Clinical Data

Patients/ Subgroups	MOG Titer fixed CBA (1:) medain(IQR) n=21	MOG Titer live CBA (1:) median(IQR) n=12	interval MRI-attack (month) median(IQR) n=23	interval NPE-attack (month) median(IQR) n=23	Interval Cortison-MRI (months) median (IQR) n=23	oral prednisolon reduction n=23
**ALL MOG**	32 (10-2109)	320 (160-640)	14,6 (5,2-30,7)	12,7 (3,2-30,7)	4 (0-13,25)	n=4
**MOG_Ci**	32(10-320)	1600 (160-5120)	688 (339-2262)	606 (169-2508)	8,5 (2-60)	n=1
**MOG_Cp**	32(10-320)	160 (0-320)	450 (134-545)	236 (48-572)	0,5 (0-18)	n=3

Abbreviations:
NPE= Neuropsychological Examination
TAP= Test of Attentional Performance
RWT= Regensburger Word fluency test
WMS-R= Wechsler Memory Scale revised
VLMT= Verbal learning and memory test
LPS= Performance Testing System
ADEM= Acute Disseminated Encephalomyelitis
ON= Optic neuritis
CBA= cell-based assay
IQR= interquartile range

MOG-antibody diagnostic was based on an established cell-based assay (MOG IFT, EUROIMMUN, Laboratory Stöcker, Germany) with a cut-off of 1:10 and on MOG Live-Cell-Assay, (Laboratory Krone, Germany) with a cut-off of 1:160).

Disease duration (DD) and Global MRI Data
To account for the significant difference in DD between MOG_Ci and MOG_Cp, we analyzed the respective correlations of global volumes and cortical thickness with regard to DD in the entire MOGAD group and the subgroups using Spearman correlations.

Table S2 Spearman correlation between disease duration and global brain volumes

	Disease duration (month) Median (IQR)		GM%/TIV	WM%/TIV	Cortical Thickness
MOGAD n=23	18 (3,25-84)	Spearman correlation coefficient	-0,309	-0,002	-0,113
		ρ	0,152	0,991	0,608
MOG_Ci n=10	9 (1-15)	Spearman correlation coefficient	-0,328	0,286	-0,103
		ρ	0,354	0,424	0,776
MOG_Cp n=13	49 (10,25-97)	Spearman correlation coefficient	-0,349	-0,193	-0,327
		ρ	0,242	0,528	0,275

Abbreviations:
GM= grey matter (calculated as percentages of total intracranial volume (TIV))
WM= white matter (calculated as percentages of total intracranial volume (TIV))

We could not verify statistically significant ($p<0.05$) correlations between DD and global brain volumes and cortical thickness. The correlation coefficient in the MOGAD group indicates a trend with regard to the correlation between DD and GM%/TIV, which should be taken into account in future analyses with higher patient numbers in the case of different disease durations.

Table S3 (A.-D.): ROI-based surface analysis showed congruent regions (marked in the same color) of significant group differences identified with the Holm-Bonferroni correction and FDR correction. Group comparisons were made for A. (All_MOGAD patients versus healthy controls), B. (MOGAD patients with cognitive impairment versus cognitive preserved MOGAD patients), C. (MOGAD patients with cognitive impairment versus healthy controls),D. (cognitive preserved MOGAD patients versus healthy controls).

A.

All_MOG versus HC											
left hemisphere (P<0.05, **uncorrected**):				left hemisphere (P<0.05, **FDR corrected**):				left hemisphere (P<0.05, **Holm-Bonferroni corrected**):			
P-value	T-value	Ze-value	aparc_DK40	P-value	T-value	Ze-value	aparc_DK40	P-value	T-value	Ze-value	aparc_DK40
0.000332	3.568.318	3.403.988	pericalcarine	0.023907	3.568.318	3.403.988	pericalcarine	0.010456	3.568.318	3.403.988	pericalcarine
0.001621	3.051.934	2.943.804	lingual	0.025130	3.051.934	2.943.804	lingual				
0.003475	2.783.830	2.699.213	inferiortemporal	0.035745	2.783.830	2.699.213	inferiortemporal				
0.004810	2.664.549	2.589.219	fusiform	0.038478	2.664.549	2.589.219	fusiform				
0.006091	2.575.714	2.506.846	lateralorbitofrontal	0.043853	2.575.714	2.506.846	lateralorbitofrontal				
0.008550	2.444.554	2.384.541	rostralanteriorcingulate								
0.013189	2.270.067	2.220.611	medialorbitofrontal								
0.013977	2.246.042	2.197.933	posteriorcingulate								
0.015280	2.208.824	2.162.754	caudalanteriorcingulate								
0.029051	1.927.449	1.894.927	cuneus								
0.036305	1.823.583	1.795.274	parstriangularis								
0.037290	1.810.863	1.783.042	parsopercularis								
0.040367	1.772.834	1.746.438	isthmuscingulate								
0.040544	1.770.720	1.744.402	temporalpole								
0.045385	1.715.703	1.691.351	superiorfrontal								
right hemisphere (P<0.05, **uncorrected**):				left hemisphere (P<0.05, **FDR corrected**):				left hemisphere (P<0.05, **Holm-Bonferroni corrected**):			
P-value	T-value	Ze-value	aparc_DK40	P-value	T-value	Ze-value	aparc_DK40	P-value	T-value	Ze-value	aparc_DK40
0.001420	3.097.102	2.984.640	medialorbitofrontal	0.025130	3.097.102	2.984.640	medialorbitofronta	0.031230	3.097.102	2.984.640	medialorbitofrontal
0.001434	3.093.762	2.981.624	fusiform	0.025130	3.093.762	2.981.624	fusiform	0.046875	2.199.438	2.153.873	lateralorbitofrontal
0.001745	3.026.647	2.920.895	middletemporal	0.025130	3.026.647	2.920.895	middletemporal				
0.002775	2.864.533	2.773.229	pericalcarine	0.033302	2.864.533	2.773.229	pericalcarine				
0.004803	2.665.094	2.589.723	inferiortemporal	0.038478	2.665.094	2.589.723	inferiortemporal				
0.010497	2.363.007	2.308.098	lingual								
0.015625	2.199.438	2.153.873	lateralorbitofrontal								
0.035605	1.832.800	1.804.134	superiorfrontal								
0.045175	1.717.983	1.693.552	temporalpole								

B.

MOG_CI versus MOG_Cp											
left hemisphere (P<0.05, **uncorrected**):				left hemisphere (P<0.05, **FDR corrected**):				left hemisphere (P<0.05, **Holm-Bonferroni corrected**):			
P-value	T-value	Ze-value	aparc_DK40	P-value	T-value	Ze-value	aparc_DK40	P-value	T-value	Ze-value	aparc_DK40
0.000381	4.002.792	3.366.487	temporalpole	0.027408	4.002.792	3.366.487	temporalpole	0.002665	4.002.792	3.366.487	temporalpole
0.013903	2.382.341	2.200.004	precuneus								
0.020369	2.195.629	2.046.182	lingual								
0.021216	2.175.380	2.029.262	insula								
0.022143	2.154.040	2.011.379	superiortemporal								
0.022989	2.135.256	1.995.595	parsopercularis								
0.023552	2.123.108	1.985.366	cuneus								
0.030781	1.986.766	1.869.435	lateraloccipital								
0.038366	1.871.652	1.769.966	transversetemporal								
0.045432	1.781.258	1.690.859	precentral								
0.048611	1.744.529	1.658.472	middletemporal					0.048611	1.744.529	1.658.472	middletemporal
right hemisphere (P<0.05, **uncorrected**):				left hemisphere (P<0.05, **FDR corrected**):				left hemisphere (P<0.05, **Holm-Bonferroni corrected**):			
P-value	T-value	Ze-value	aparc_DK40								
0.008076	2.639.756	2.405.466	superiortemporal								
0.015315	2.335.561	2.161.840	temporalpole								
0.015341	2.334.734	2.161.163	lingual								
0.035912	1.906.504	1.800.232	medialorbitofrontal								
0.046049	1.773.952	1.684.428	posteriorcingulate								
0.046344	1.770.499	1.681.386	caudalanteriorcingulate								

C.

MOG_CI versus HC											
left hemisphere (P<0.05, **uncorrected**):				left hemisphere (P<0.05, **FDR corrected**):				left hemisphere (P<0.05, **Holm-Bonferroni corrected**):			
P-value	T-value	Ze-value	aparc_DK40	P-value	T-value	Ze-value	aparc_DK40	P-value	T-value	Ze-value	aparc_DK40
0.000183	3.798.589	3.563.700	lingual	0.005727	3.798.589	3.563.700	lingual	0.006033	3.798.589	3.563.700	lingual
0.000239	3.714.666	3.493.215	pericalcarine	0.005727	3.714.666	3.493.215	pericalcarine	0.010499	3.714.666	3.493.215	pericalcarine
0.000925	3.271.716	3.113.419	fusiform	0.012338	3.271.716	3.113.419	fusiform	0.029589	3.271.716	3.113.419	fusiform
0.001127	3.204.269	3.054.465	cuneus	0.012338	3.204.269	3.054.465	cuneus				
0.001371	3.136.900	2.995.288	inferiortemporal	0.012338	3.136.900	2.995.288	inferiortemporal	0.016451	3.136.900	2.995.288	inferiortemporal
0.001874	3.027.614	2.898.683	parsopercularis	0.013490	3.027.614	2.898.683	parsopercularis				
0.003217	2.833.003	2.724.826	temporalpole	0.018771	2.833.003	2.724.826	temporalpole	0.045035	2.833.003	2.724.826	temporalpole
0.003610	2.790.484	2.686.535	lateralorbitofrontal	0.018771	2.790.484	2.686.535	lateralorbitofrontal	0.018049	2.790.484	2.686.535	lateralorbitofrontal
0.003718	2.779.586	2.676.704	precentral	0.018771	2.779.586	2.676.704	precentral				
0.003911	2.760.749	2.659.693	parstriangularis	0.018771	2.760.749	2.659.693	parstriangularis				
0.004426	2.714.401	2.617.751	precuneus	0.019322	2.714.401	2.617.751	precuneus				
0.004562	2.702.940	2.607.359	middletemporal	0.019322	2.702.940	2.607.359	middletemporal				
0.005156	2.656.485	2.565.161	rostralanteriorcingulat	0.020253	2.656.485	2.565.161	rostralanteriorcingulate	0.005156	2.656.485	2.565.161	rostralanteriorcingulate
0.005344	2.642.804	2.552.711	superiortemporal	0.020253	2.642.804	2.552.711	superiortemporal				
0.009343	2.423.559	2.351.725	paracentral	0.030396	2.423.559	2.351.725	paracentral				
0.017296	2.166.884	2.113.100	posteriorcingulate	0.042988	2.166.884	2.113.100	posteriorcingulate				
0.018604	2.135.266	2.083.468	lateraloccipital	0.044650	2.135.266	2.083.468	lateraloccipital				
0.021212	2.077.636	2.029.332	postcentral	0.045980	2.077.636	2.029.332	postcentral				
0.021713	2.067.290	2.019.595	insula	0.045980	2.067.290	2.019.595	insula				
0.023398	2.033.902	1.988.139	transversetemporal	0.046796	2.033.902	1.988.139	transversetemporal				
0.032937	1.876.658	1.839.280	rostralmiddlefrontal								
0.034149	1.859.577	1.823.041	superiorfrontal								
0.040574	1.776.700	1.744.062	medialorbitofrontal								
0.041492	1.765.774	1.733.628	caudalanteriorcingulate								
right hemisphere (P<0.05, **uncorrected**):				left hemisphere (P<0.05, **FDR corrected**):				left hemisphere (P<0.05, **Holm-Bonferroni corrected**):			
P-value	T-value	Ze-value	aparc_DK40	P-value	T-value	Ze-value	aparc_DK40	P-value	T-value	Ze-value	aparc_DK40
0.000155	3.850.973	3.607.454	medialorbitofrontal	0.005727	3.850.973	3.607.454	medialorbitofrontal	0.007730	3.850.973	3.607.454	medialorbitofrontal
0.000402	3.547.387	3.351.310	lingual	0.007239	3.547.387	3.351.310	lingual	0.010456	3.547.387	3.351.310	lingual
0.001343	3.144.150	3.001.671	pericalcarine	0.012338	3.144.150	3.001.671	pericalcarine				
0.001831	3.035.795	2.905.941	superiortemporal	0.013490	3.035.795	2.905.941	superiortemporal				
0.002378	2.942.644	2.823.060	fusiform	0.015568	2.942.644	2.823.060	fusiform				
0.006022	2.596.877	2.510.834	middletemporal	0.021680	2.596.877	2.510.834	middletemporal				
0.008643	2.454.832	2.380.559	lateralorbitofrontal	0.029634	2.454.832	2.380.559	lateralorbitofrontal				
0.009710	2.408.022	2.337.381	inferiortemporal	0.030396	2.408.022	2.337.381	inferiortemporal				
0.011125	2.352.571	2.286.079	transversetemporal	0.033374	2.352.571	2.286.079	transversetemporal				
0.011595	2.335.531	2.270.281	temporalpole	0.033394	2.335.531	2.270.281	temporalpole				
0.014063	2.255.130	2.195.531	cuneus	0.038943	2.255.130	2.195.531	cuneus				
0.015982	2.200.825	2.144.852	parsorbitalis	0.042620	2.200.825	2.144.852	parsorbitalis				
0.017315	2.166.423	2.112.668	caudalanteriorcingulat	0.042988	2.166.423	2.112.668	caudalanteriorcingulate				
0.020175	2.099.779	2.050.152	precentral	0.045980	2.099.779	2.050.152	precentral				
0.021310	2.075.606	2.027.422	posteriorcingulate	0.045980	2.075.606	2.027.422	posteriorcingulate				
0.022625	2.048.951	2.002.324	postcentral	0.046543	2.048.951	2.002.324	postcentral				
0.025736	1.990.878	1.947.526	paracentral								
0.031945	1.891.049	1.852.951	inferiorparietal								
0.033472	1.869.052	1.832.050	supramarginal								
0.040500	1.777.588	1.744.911	superiorfrontal								

D.

MOG_Cp versus HC											
left hemisphere (P<0.05, **uncorrected**):				left hemisphere (P<0.05, **FDR corrected**):				left hemisphere (P<0.05, **Holm-Bonferroni corrected**):			
P-value	T-value	Ze-value	aparc_DK40					P-value	T-value	Ze-value	aparc_DK40
0.022136	2.056.209	2.011.510	pericalcarine					0.044272	2.056.209	2.011.510	pericalcarine
right hemisphere (P<0.05, **uncorrected**):				left hemisphere (P<0.05, **FDR corrected**):				left hemisphere (P<0.05, **Holm-Bonferroni corrected**):			
P-value	T-value	Ze-value	aparc_DK40								
0.010884	2.357.990	2.294.402	middletemporal								
0.030966	1.903.564	1.866.776	fusiform								
